# Hemodialysis Catheter-Related Thrombi: A Challenging Patient

**DOI:** 10.7759/cureus.8438

**Published:** 2020-06-04

**Authors:** Catarina Abrantes, Elsa Soares, Patrícia Valério, Teresa Furtado, Carlos Barreto

**Affiliations:** 1 Nephrology, Setubal Hospital Center, Setubal, PRT; 2 Nephrology, Centro Hospitalar De Setubal, Setubal, PRT

**Keywords:** hemodialysis catheter, multiple myeloma, atrial thrombus

## Abstract

Patients requiring renal replacement therapy remain a significant burden on the healthcare system. A substantial amount of hospitalization costs for these patients are related to vascular complications, especially catheter-related thrombosis, which is associated with a significant increase in morbidity and mortality.

We report the case of a male patient with multiple myeloma (MM) and dialysis-dependent renal failure due to light-chain cast nephropathy, who presented recurrent early catheter dysfunction. A large thrombus was detected, extending from the superior vena cava (SVC) to the right atrium (RA) and later at the inferior vena cava (IVC), ultimately leading to exhaustion of vascular capital.

To this date, there is limited evidence regarding the best approach to catheter-related thrombosis, which frequently leads to treatment dilemmas in clinical practice and demands careful evaluation and individualized decisions. In patients with a highly thrombotic profile, peritoneal dialysis may be considered earlier to prevent further vascular capital damage.

## Introduction

Renal replacement therapy, although lifesaving, imposes heavy costs for hospitals and complications arising from the patients' vascular access are often the cause. Although not considered the preferred method for hemodialysis (HD), central venous catheters (CVCs) remain a necessity in both incident and prevalent HD patients. Catheter-related complications include infections, catheter occlusion, and central vein stenosis [1]. Catheter-related thrombosis is a potentially serious problem of central venous catheterization in renal failure patients, generally associated with an increase in morbidity and mortality. Management of this entity remains controversial, due to the lack of specific data [2].

## Case presentation

A 56-year-old male patient with newly diagnosed multiple myeloma (MM) was admitted to the Nephrology Department of our hospital following dialysis-dependent renal failure due to light-chain cast nephropathy. He was receiving therapy with bortezomib, cyclophosphamide, and dexamethasone.

**Figure 1:**
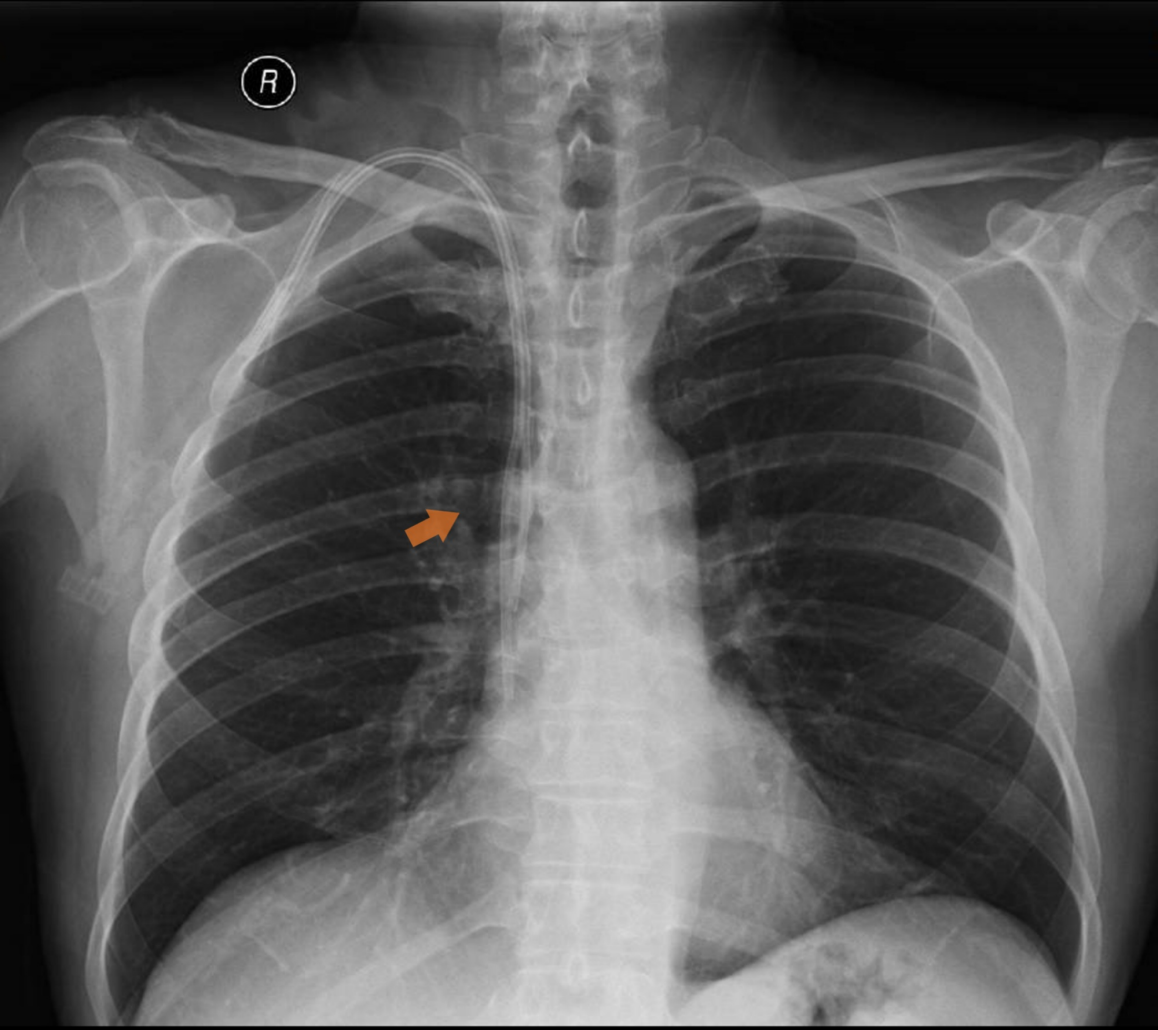
Chest X-ray.

The patient was submitted to a double-lumen polyurethane HD catheter placement in the right internal jugular (IJ) vein (GamCath®, Baxter-Gambro, Illinois, EUA, 12 Fr., 150 mm tip-to-cuff), with no immediate complications. Unfractionated heparin was administered intravenously in each dialysis session and heparin was used for catheter lock at the end to ensure catheter patency. After 11 days, CVC was replaced by a polyurethane tunneled HD catheter (Arrow® Cannon® II Plus,Teleflex, Pennsylvania, EUA, 15 Fr., 190 mm tip-to-cuff), but early dysfunction was noted, which persisted despite the use of thrombolytic treatment (tissue plasminogen activator - tPA), leading to inadequate dialysis. Chest X-ray showed correct anatomical catheter positioning, with the tip in the entrance to the right atrium (RA) and its arterial lumen facing the mediastinum (Figure 1).

Catheter replacement was carried out with angiographic support. However, after IV contrast injection, a fibrin sheath was visible surrounding the catheter’s lumen. Hence, the catheter was removed and an attempt was made to insert a new one in the same location but escaping the fibrin sheath. During guidewire insertion the patient developed sudden ventricular fibrillation and was transferred to the ICU after successful resuscitation.

**Figure 2:**
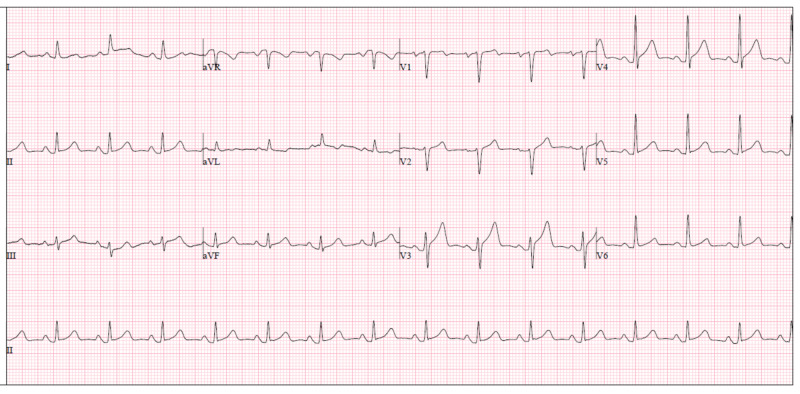
Electrocardiogram.

On admission, no abnormality was found on physical examination and there were no signs of hemodynamic instability with inotropic support at low doses. Arterial blood gases on mechanical ventilation were within normal levels (pH - 7.36, PO2 - 160 mmHg, PCO2 - 35 mmHg, and HCO3 - 19.8 mmol/L), as well as analytic workup, with the exception of hyperlactacidemia (3.7 mmol/L). Blood workup was normal and both electrocardiogram (Figure 2) and transthoracic echocardiography (TTE) showed no abnormal findings, favoring suspicion of a mechanically induced arrythmia.

**Figure 3:**
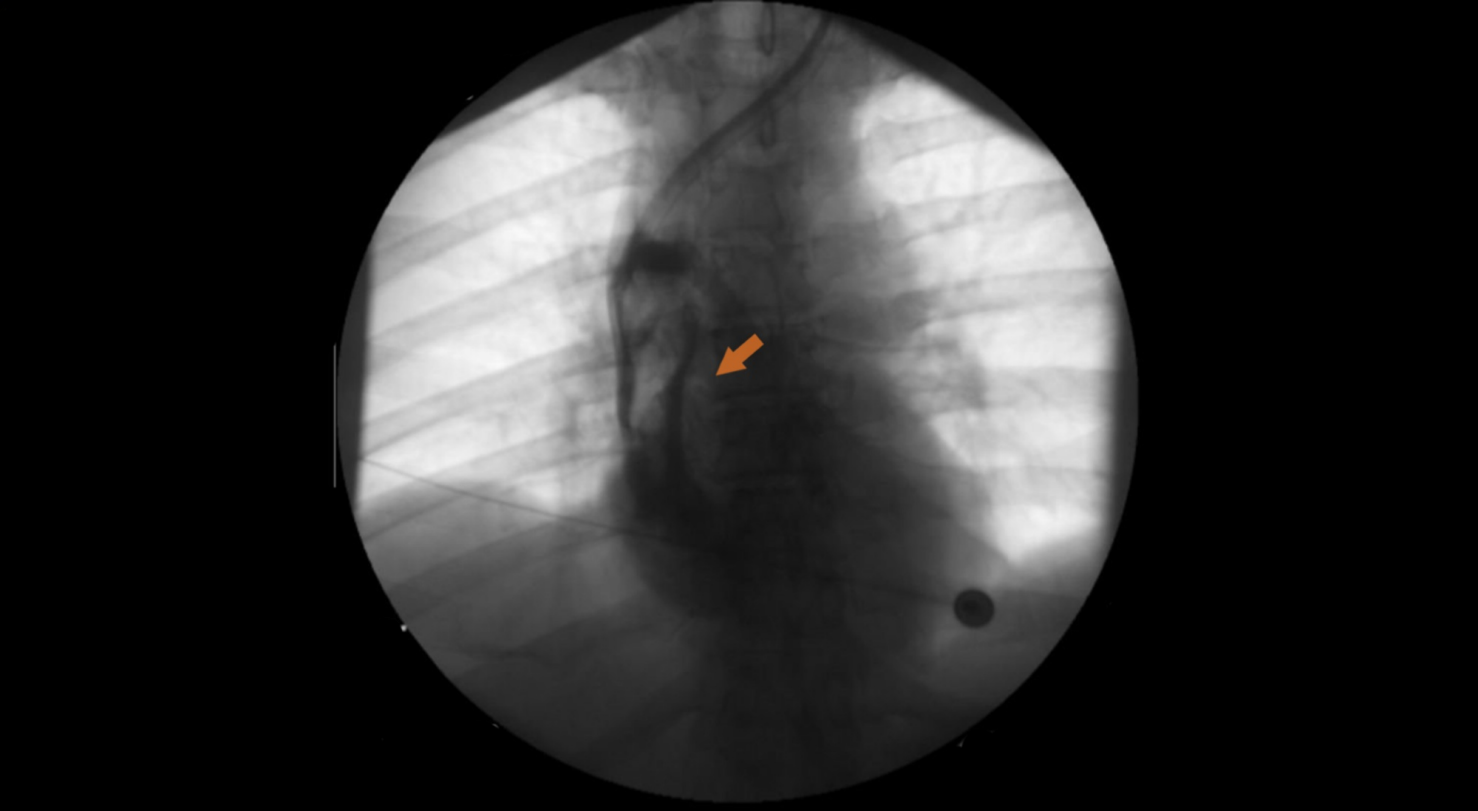
Catheter-related thrombus in SVC. Fluoroscopy showing catheter-related thrombus (arrow) in superior vena cava (SVC).

**Figure 4:**
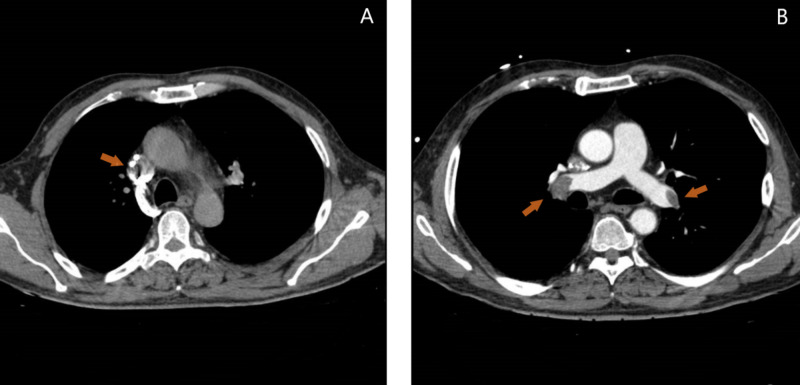
CTA findings. A: Computed tomography angiography (CTA) scan of the thorax with a nonocclusive thrombus in the superior vena cava (SVC) (arrow). B: Evidence of bilateral pulmonary thromboembolism (arrows).

The patient evolved favorably, with no signs of hemodynamic or neurologic complications. As implantation of a new HD catheter was urgent, on the following day a nontunneled catheter was inserted in the left IJ vein. However, a thrombus was now evident in the SVC (Figure 3), confirmed through computed tomography angiography (CTA) of the chest and neck, which demonstrated a nonocclusive thrombus surrounding the HD catheter and extending from the SVC to the RA (Figure 4A). Signs of bilateral pulmonary thromboembolism were also present, involving both lobar and segmental pulmonary arteries (Figure 4B). Repeat TTE did not show signs of right ventricular dysfunction. From a clinical standpoint, the patient did not appear to be in respiratory distress, with no accessory muscle use. His blood pressure was 120/71 mmHg, pulse 86 beats per minute, respiratory rate 18 breaths per minute, and oxygen saturation of 95% on room air. The lungs were clear to auscultation, and he exhibited no wheezing, rhonchi, or rales. The heart sounds were regular, with no audible murmur.

After careful multidisciplinary discussion and given the lack of symptoms or cardiovascular instability, systemic thrombolysis was not carried out. Because the surgical risk was high, vacuum-assisted thrombectomy (VAT), a negative pressure mechanical device that is used for thrombus extraction, was performed under fluoroscopy and continuous TTE guidance but was unable to remove RA and SVC thrombus. Systemic anticoagulation was initiated with low molecular weight heparin (LMWH) and later switched to warfarin. Given the high risk of intracardiac thrombus mobilization, left IJ vein HD catheter was not removed.

As catheter performance measures were suboptimal, a right-cuffed femoral vein catheter was placed (Jet Flow High Flow®, Jet Medical SA, La Chaux-de-Fonds, Switzerland, 15 Fr., 360 mm tip-to-cuff). Once again, early dysfunction was apparent, even under therapeutic levels of warfarin and after several thrombolytic schemes with tPA. For this reason, the catheter was replaced for a new one with 550 mm (tip-to-cuff), using fluoroscopy. 

**Figure 5:**
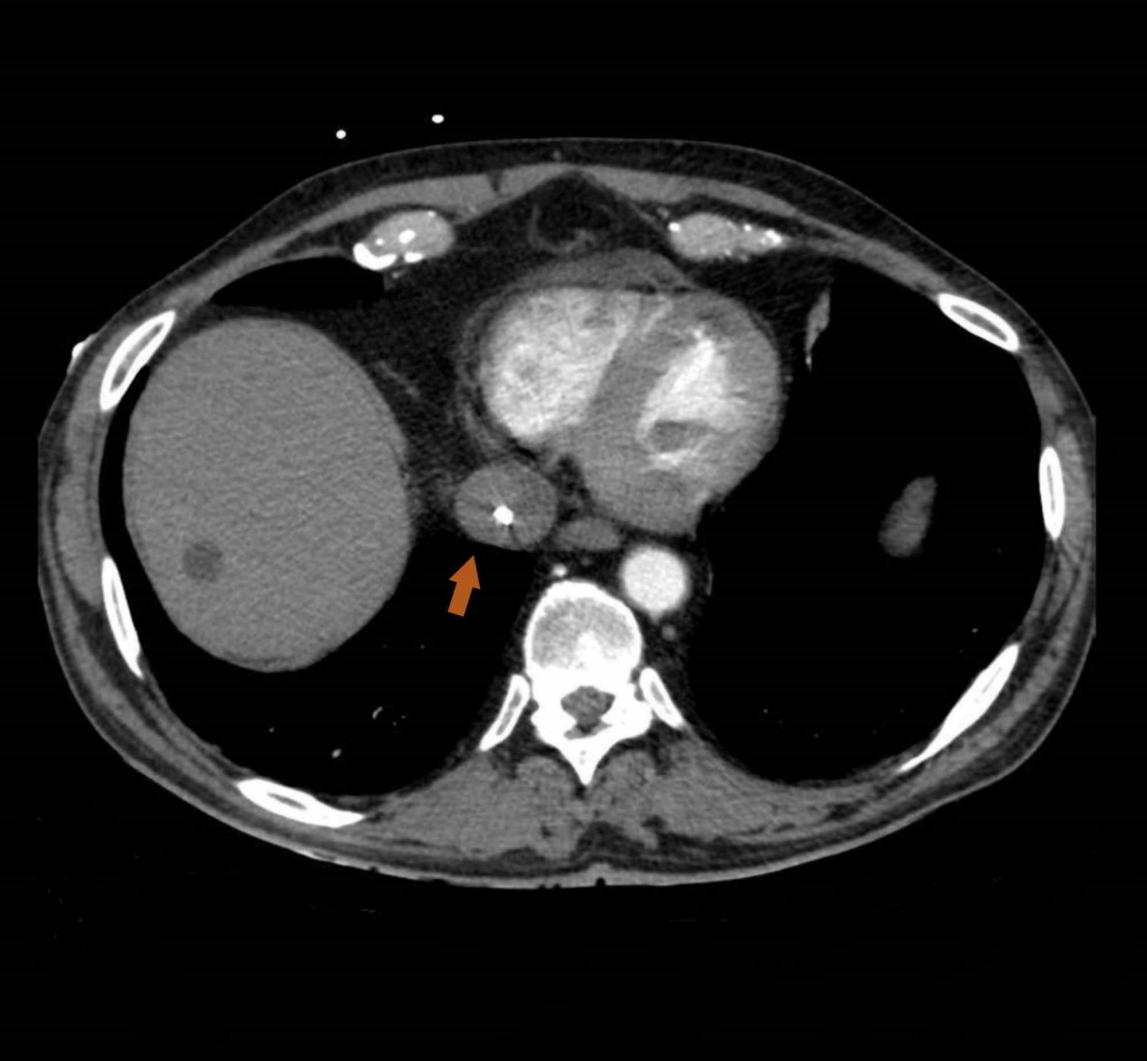
Catheter-related thrombus in IVC. CT scan showing a partial thrombus (arrow) surrounding the hemodialysis catheter in inferior vena cava (IVC).

One week later, the patient presented to the ED with signs of septic shock and a cutaneous abscess was evident in the catheter’s tunnel tract. Intravenous antibiotic therapy was started and abscess drainage was carried out, with clinical improvement. Surprisingly, catheter malfunction was noted once more. CT scan was repeated, demonstrating a new thrombus surrounding the HD catheter in inferior vena cava (IVC) with 3 cm of longitudinal extension (Figure 5). Persistent elevation of inflammatory parameters despite directed antibiotic therapy was another concern to the medical staff.

Given the need for catheter removal in a patient with vascular access failure, peritoneal dialysis (PD) was opted for as a last resort. After Tenckhoff catheter placement and removal of femoral HD catheter, the patient was switched to an urgent-start PD scheme. Meanwhile, left IJ vein HD catheter was also successfully removed, despite unresolved RA thrombus, and warfarin therapy was continued given the high thrombotic burden. Currently, after seven months on PD and regular follow-up, neither recurrent infection nor thrombotic-related events have occurred.

## Discussion

Multiple myeloma is the second most common hematologic cancer worldwide, accounting for 1% of all malignant diseases and with an annual European estimated incidence of 4.5-6.0 cases/100,000 population [3]. Renal involvement is a well-known feature of this disorder, but only a minority of patients suffer irreversible renal failure requiring chronic hemodialysis (HD) [4]. Hemostatic abnormalities are also correlated with MM, more often bleeding than thrombosis.

We presented the case of a MM patient with extensive thrombi surrounding HD catheters, the first extending from the SVC to the RA, which is a rare and likely life-threatening phenomenon [5]. Thrombus-related catheter occlusion may occur in both early and late catheter dysfunction, although more commonly associated with the latter. Intimal vessel injury, turbulent blood flow, and activation of coagulation cascades with fibrin sheath formation are major contributors to thrombus formation. In this particular case, however, a hyperviscosity state should also be considered. Hypercoagulability in MM may reflect alterations on fibrin structure due to immunoglobulins, vascular endothelial injury secondary to circulating inflammatory cytokines or tumor cells, acquired activated protein C resistance, antithrombin III deficiency, protein C or S deficiency, decreased plasminogen activity or paraproteinemia, all of which are possible in this case. Unfortunately, thrombophilia screening could not be performed as the patient was under anticoagulant therapy. Also of note is the fact that a total of four cycles of bortezomib, cyclofosfamide, and dexamethasone were administered before the thrombotic findings, at which time chemotherapy was suspended. Interestingly, the use of a proteasome inhibitor in therapeutic regimens for MM seems to protect patients from chemotherapy-induced thrombotic events [6], which raises suspicion of other possible factors at play in this case. On the other hand, despite being under erythropoietin-stimulating agents (ESA) therapy, current evidence does not support an increase in the risk of thrombotic events, although there are conflicting results [7-8].

Pulmonary or systemic embolization are tragic complications of atrial thrombi and carries a poor prognosis. Management remains challenging, especially in asymptomatic patients, as was this case [9]. Systemic thrombolysis, surgical thrombectomy, and VAT are all plausible options. However, given the limited recommendations available, the most appropriate strategy should be individualized. VAT is a catheter-based thrombectomy system that performs mechanical suction of heart or vascular thrombus, and is indicated for patients who are poor candidates for surgery and/or have a contraindication to thrombolysis. It consists of a 22F venous catheter (suction cannula), inserted percutaneously or via surgical cut-down, and can remove thrombi using a centrifugal pump and a venous reinfusion cannula. Successful percutaneous aspiration has been reported [10], yet, in our patient the thrombus was already organized. Despite catheter removal being advised, the risk of thrombus detachment and embolization must be weighed. In our case, systemic anticoagulation therapy was initiated, as advocated by available evidence [11], however, uncertainty regarding the timing for catheter removal made clinical decisions even harder.

## Conclusions

In conclusion, there is limited evidence for the best approach to catheter-related thrombi, leading to treatment dilemmas in routine clinical practice. The case presented is impressive for the unique thrombotic profile that ultimately led to an exhaustion of vascular capital, with a final favorable outcome after transition to PD, raising the question if in similar situations PD should be considered earlier. In sum, we highlight the need for careful evaluation and individualized decisions in these patients.
